# The Role of Malting and Brewer’s Spent Grain in Sustainable Cereal Utilization

**DOI:** 10.3390/foods15020287

**Published:** 2026-01-13

**Authors:** Szintia Jevcsák, Gerda Diósi, Gréta Törős, Ádám Fülep, Endre Máthé

**Affiliations:** 1 Institute of Food Technology, Faculty of Agricultural and Food Sciences and Environmental Management, University of Debrecen, Böszörményi Street 138, 4032 Debrecen, Hungary; diosi@agr.unideb.hu; 2 Institute of Animal Science, Biotechnology and Nature Conservation, Faculty of Agricultural and Food Sciences and Environmental Management, University of Debrecen, Böszörményi Street 138, 4032 Debrecen, Hungary; 3 Doctoral School of Animal Husbandry, Faculty of Agricultural and Food Sciences and Environmental Management, University of Debrecen, Böszörményi Street 138, 4032 Debrecen, Hungary; 4 Institute of Nutrition, Faculty of Agricultural and Food Sciences and Environmental Management, University of Debrecen, Böszörményi Street 138, 4032 Debrecen, Hungary; fulep.adam@icloud.com (Á.F.); endre.mathe@agr.unideb.hu (E.M.); 5 Institute of Life Sciences, Faculty of Medicine, Vasile Goldis Western University of Arad, 310414 Arad, Romania

**Keywords:** sustainable malting, grains, nutritives, bioactive compounds, functional properties, quality control

## Abstract

Malting is a sustainable, low-cost, and adaptable technique that enhances the nutritional and functional value of cereals while contributing to waste reduction, improved food safety, and the valorization of brewing by-products such as brewers’ spent grain. It was originally developed for barley but is now used with a wide range of cereals. Malting, in its simplest form, involves controlled germination and drying, which enhance enzyme activity and improve grain nutritional quality. Our review introduces a broader perspective by addressing how malting can enhance health benefits through malted forms of both common and less prominent cereals such as sorghum, teff, millet, triticale, quinoa, and buckwheat. Nutritional enhancement takes place by increasing nutrient bioavailability, changing chemical composition, and reducing antinutrients, while inducing the production of bioactive compounds with antioxidant, anti-inflammatory, and antidiabetic activities. This review examines brewers’ spent grain (BSG), a nutrient-dense brewing by-product that is widely recognized as a sustainable ingredient for food and nutrition applications.

## 1. Introduction

Cereal crops account for approximately 73% of the world’s harvested agricultural land and contribute nearly 60% of global food production [1,2]. In the context of rapid global population growth, food security and long-term nutritional sustainability have become increasingly important. Consequently, enhancing the nutritional value and versatility of cereal-based foods is a major research priority [3].

Malting involves a controlled germination process followed by kilning. This traditional technique alters the physical and biochemical structure of grains, activating natural enzymes and promoting the development of beneficial compounds [4,5,6]. As a result, malting induces favorable biochemical changes that can enhance the nutritional and functional properties of cereals [5]. Malt-derived products, including both alcoholic and non-alcoholic fermented beverages, have been associated with a range of potential health-promoting effects [4,5,6,7,8].

In recent years, researchers have become interested in brewers’ spent grain (BSG), which is a primary by-product of the brewing process. This material represents approximately 85% of total brewery by-products, with an estimated global annual production of around 39 million tons. Although BSG is rich in fiber, protein, lysine, polyphenols, and various micronutrients, it remains underutilized in human diets. Remarkably, even after the brewing process, BSG still retains valuable bioactive compounds, such as β-glucans and ferulic acid [3,4]. The nutritional value of bread, snacks, and beverages can be enhanced by the incorporation of BSG, while the amount of waste can also be reduced. The re-utilization of these products can align with the principles of a circular economy, supporting zero-waste innovations [5,6].

Malting plays a key role in increasing the diversity of phytochemicals in cereals during germination, which typically lasts between 50 and 96 h. Moisture and oxygen stimulate metabolic activities, which, in turn, increase enzyme production and initiate the breakdown of macronutrients. This process also results in the formation of beneficial bioactive compounds [6]. Once the kilning step occurs, it halts enzyme activity and preserves these compounds, making malted cereals ideal for functional foods that go beyond just brewing [7,8].

Despite extensive research on malting processes and brewing by-products, an integrated evaluation of malting-induced nutritional changes alongside the upcycling potential of brewers’ spent grain for sustainable human food applications remains limited.

This review addresses the following research questions: How does malting influence the nutritional, functional, and health-related properties of cereals? And how can brewer’s spent grain be valorized as a sustainable, safe, and biofunctional ingredient within circular cereal-based food systems?

## 2. Methodology of the Review

The growing interest in cereal malting and sustainable valorization of brewers’ spent grain (BSG) reflects the need to reduce nutrient pollution, manage by-products, and create value from agri-food waste. This narrative review was selected due to the highly heterogeneous nature of the literature, which varies widely in cereal type, malting conditions, analytical methods, and applications, making quantitative meta-analysis impractical. The narrative approach allows for an integrated interpretation of the biochemical, nutritional, functional, and sustainability factors relevant to circular cereal-based food systems.

The review addresses four main questions: (A) how malting alters the nutritional and functional properties of cereals compared with germination alone; (B) which biochemical transformations during malting enhance health-related attributes; (C) how BSG can be valorized as a safe, sustainable, and biofunctional ingredient; and (D) what technological and sustainability constraints limit its large-scale use.

Literature searches were conducted in Scopus, Web of Science, PubMed, and ScienceDirect using terms related to malting, cereal germination, kilning, BSG, cereal by-products, functional foods, and circular economy. Studies published between 2015 and 2025 were prioritized, with earlier key papers included for background. Additional references were identified through citation tracking.

**Figure 1:**
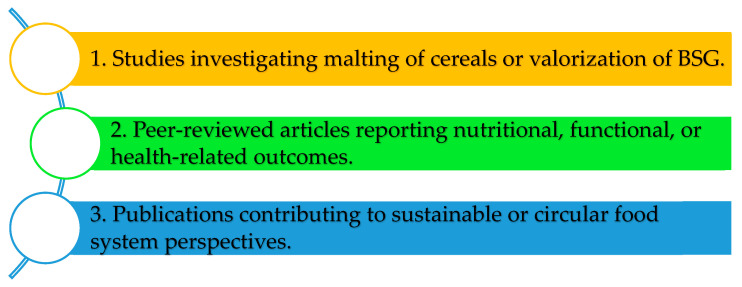
Selected inclusion criteria for this study.

Studies were selected according to predefined inclusion criteria (Figure 1). As this is a narrative review, no formal PRISMA-based screening or risk-of-bias assessment was applied; instead, studies were chosen based on relevance, methodological quality, and contribution to the review objectives.

Study selection followed three main inclusion criteria, illustrated in Figure 1.

## 3. Cereals and Raw Materials for Malting

Choosing the right raw materials is a crucial first step in the malting process as it has a direct impact on the safety, quality, and overall effectiveness of the malt produced. It is not just about picking the right cereal types or grain mixes. Selecting the right cereal types or grain mixes is important, but it is equally vital to ensure the grains are free from foreign matter, microbial contamination, and chemical residues [9]. These factors are crucial for achieving consistent germination and ensuring food safety. During harvesting, storage, and transportation, cereals are exposed to physical, chemical, and biological risks that can reduce their viability. Quality assurance is highly important throughout the entire supply chain [5,10].

The cleaning of the initial grain, water purity, uniformity in size and shape, and biochemical markers such as germination energy, protein and starch content, test weight, and extract yield are highly important, resulting in high-quality product. Laboratory testing is also essential in processing [6,11]. Selecting the best raw materials helps facilitate uniform steeping, effective enzymatic activation, and stable compound formation during kilning, contributing to fermentation and high nutrition.

Barley (*Hordeum vulgare*) is the dominant malting grain due to its high starch content and natural husk, which facilitate processing. Its adaptability also makes it relevant in addressing climate challenges, meeting diverse dietary preferences, and supporting agricultural diversification. Some examples are wheat, rye, oats, triticale, millet, sorghum, and, more recently, pseudocereals (e.g., quinoa, buckwheat, amaranth), as well as legumes such as lentils and chickpeas [12,13], with unique technological characteristics such enzyme activity profiles, β-glucan content, and diastatic power. Each alternative grain requires tailored malting protocols to optimize enzymatic activity, starch conversion, and flavor development during brewing.

Einkorn, teff, and tritordeum have recently gained attention for their potential sustainability benefits. For example, teff is drought-tolerant and nutrient-rich, making it a suitable gluten-free option that can support diversified cropping systems [14]. Tritordeum is a hybrid of *Hordeum chilense* and *Triticum turgidum* and also a malting grain, valued for its favorable nutritional and agronomic properties [15,16].

A comparative study revealed that tritordeum malt exhibits lower grain glassiness and better grading than barley, which improves uniform steeping and enzymatic accessibility during malting. Furthermore, beers produced from 100% tritordeum malt achieve high-quality characteristics comparable to conventional barley-based brews, demonstrating the practical feasibility of using this alternative grain in commercial brewing. Table 1 and Table 2 provide a comparative overview of the malt parameters for both grains [17].

Process optimization is increasingly important. Steeping duration, germination time, and kilning profiles must be carefully adjusted for each alternative grain to maximize enzymatic activity and compound formation. These adjustments require up-to-date, advanced malting technologies. Process optimization plays a role in overall enzymatic activity and the formation of beneficial compounds, including flavonoids, antioxidants, and polyphenols. It can be said that the diversification of the raw materials used in malting further supports the range of functional ingredients available. By diversifying the grains used in malting, these alternative crops can enhance agricultural resilience and contribute to more circular food systems, for instance, through improved resource use efficiency and adaptation to local growing conditions.

## 4. The Malting Process: Biochemical Transformation of Cereal Grains

Malting is among the oldest known applications of biotechnology in food production. It converts raw cereal kernels into malt, a nutritionally enriched and enzymatically active material, designed for fermentation or functional food manufacturing. Although barley remains the primary grain used, growing demand for gluten-free, climate-resilient, and functionally improved cereals has expanded its application to other grain types. This bioconversion comprises three consecutive phases: steeping, germination, and kilning, each facilitating distinct physiological and biochemical changes [19]. Throughout these steps, endogenous enzymes are either activated or newly synthesized, catalyzing the breakdown of complex macromolecules, including starch, proteins, and non-starch polysaccharides. Simultaneously, bioactive substances like phenolic compounds and antioxidants are generated [20].

Mass balance studies have shown that up to 80% of the original dry mass of the grain undergoes metabolic reconfiguration, resulting in a substrate with improved fermentation efficiency and superior nutritional properties [21]. Beyond its biochemical role, malting is central to the preparation of fermented beverages such as beer. In barley-based workflows, the typical sequence includes soaking, sprouting, and drying, which collectively generate several by-products, including husks, draff (wet spent grain), and steep liquor. The volume and nature of these residuals depend on both the type of cereal and operational parameters [22].

BSG, generated during industrial brewing, typically arises after the malting and mashing stages, which, together, last several days. This by-product constitutes approximately 70–80% of the residual dry biomass and has been studied for valorization, such as recovery of functional compounds from the husk and reuse of remaining solids in animal nutrition or baked goods [23,24].

The malting cycle is initiated by soaking cereal grains in water to activate the seeds, enhance respiratory processes, and initiate key metabolic pathways. During this phase, the internal moisture content of the grain rises to approximately 40%, while alternating oxygen-rich (aerobic) conditions support efficient oxidative metabolism. After 36 to 48 h of hydration, the moistened grains are transferred to germination units, where controlled sprouting continues for an additional 24 to 48 h. Germination is then interrupted by a carefully managed drying process, reducing moisture to below 5% to ensure product stability. This final stage, known as kilning, involves a gradual increase in temperature tailored to the specific malt type being produced. Depending on the cereal species and the particular process parameters, the complete malting procedure typically spans 1 to 3 weeks [19].

### 4.1. Steeping

Steeping initiates the malting process by increasing the grain’s moisture content from around 12–14% to 42–47%. This moisture is necessary to trigger metabolic activity, including enzyme synthesis, respiration, and the breakdown of seed dormancy. Alternating wet and dry cycles provides oxygenation and avoids anaerobic stress, promoting uniform hydration. The steeping duration is typically 24–48 h, depending on the grain type and desired malt characteristics [19,25,26].

### 4.2. Germination

Germination follows steeping and lasts between 3 and 5 days under controlled conditions. The primary goal is enzymatic mobilization, particularly through the action of α-amylase, β-amylase, proteases, and cellulases, which convert starches and proteins into fermentable and soluble fractions. Structural transformations during germination include radicle emergence, protein solubilization, and partial cell wall breakdown [19].

Grain variety, temperature, and germination time all influence enzymatic activity. For example, germinating tritordeum for extended periods has been shown to enhance enzyme profiles, thereby improving its functional properties, although detailed data are presented in the raw materials section [16].

### 4.3. Kilning

Kilning is the final phase, where germination is halted and the malt is stabilized. The grain is dried in staged temperature increments, reducing moisture to 4–5%, inactivating unwanted enzymes, and encouraging Maillard reactions that develop color, flavor, and aroma. The challenge is to preserve enzymatic activity, critical to fermentation, while achieving the desired sensory attributes. Depending on the final kilning temperature and profile, malts can have distinct flavor profiles, such as pale, Vienna, or Munich. Properly kilned malt is shelf-stable for up to 12 months without significant quality loss, making it a versatile and valuable ingredient across various food and beverage applications [27].

## 5. Malt Types and Products

**Figure 2:**
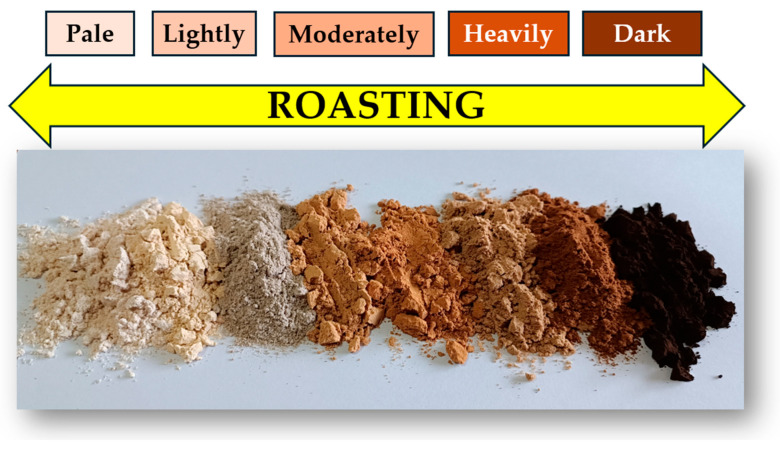
A wide range of colors could emerge during the roasting process of malting.

Malt exists in a wide variety of types and products (Figure 2), but pale lager malt remains the benchmark in brewing due to its high extract and strong enzymatic activity. Its gentle kilning (≤85 °C) preserves enzymes critical for starch conversion, making it essential for efficient fermentation, despite contributing minimal color or flavor [28].

Pale ale malt, commonly used in top-fermented beers, provides greater color and flavor than pale lager malt due to slightly higher kilning temperatures (~90 °C) [29]. Vienna malt, kilned under similar conditions with recirculated air, develops a reddish hue and caramel notes. These kilning-induced chemical changes illustrate how malt processing can be tailored for specific sensory outcomes in beer production [30].

Munich malt, kilned at 100–105 °C, develops a deep color and intense aroma due to partial over-modification during germination. While enzymatic activity is reduced, these malts are valued for flavor contribution rather than fermentable sugar content, highlighting the trade-off between sensory attributes and biotechnological utility [4].

Malt exists in a wide variety of types and products, each developing distinct color profiles during roasting, as illustrated in Figure 2.

Caramel (crystal) and Carapils malts, fully modified and initially roasted at 65–70 °C, are further heated (crystal malt: 150–180 °C) to generate characteristic caramel flavors and color. This controlled roasting sacrifices enzyme activity but enhances sweetness and visual appeal, illustrating the balance between functional and sensory properties in malt selection [30].

Dark malts, including Brown, Amber, Chocolate, and Black malts, are roasted at high temperatures (100–220 °C), producing intense color and flavor while eliminating enzymatic activity [4]. These malts are primarily used for sensory impact rather than fermentation efficiency, highlighting the inverse relationship between enzyme retention and flavor intensity in malt processing [31].

Wheat malt, the second most common malted grain, provides high extract with low tannin content, reducing haze in beer. Beyond brewing, germinated wheat imparts natural sweetness in food applications, linking malting techniques to both sensory quality and functional performance [32].

Sorghum malt is used to make a traditional African beer, called kaffir, which is widely used in commercial malt production, particularly in Africa. However, sorghum germination produces few enzymes, resulting in poor endosperm modification and greater malt losses [23].

Other cereals used for malting include rye, oats, and triticale. Rye malt is traditionally used in whiskey production, offering high enzyme and extract content, as well as viscosity. The use of oat malt has declined due to its high lipid content, which can lead to taste issues. Triticale malt has a strong flavor but also presents high viscosity issues [24].

Syrups and extracts are often produced from malts and used in the early stages of brewing. They retain their color, sweetness, and flavor but lose enzymatic activity, requiring supplementation with wheat flour [33].

Malts are widely used in the food industry (e.g., malt distilling, grain distilling, brewing beer, baking), where various malt types are selected based on their specific color, flavor, or enzymatic properties [31].

Perhaps the best-known use of malt is in beer production [34]. The raw material for brewing is enzyme-active malt, while the by-product brewer’s spent grain is enzyme-inactive [6]. Extensive research is being conducted into how brewers’ spent grain, which is generated in large quantities during beer production, can be repurposed in food development [35].

Recent research has shifted malt characterization from descriptive traits to mechanistic insights (Table 3). Kilning temperature influences Maillard intermediates, driving flavor and color formation, while germination duration determines enzymatic potential. Malt-derived enzymes now enable functional applications, such as gluten reduction, without compromising sensory quality. These findings demonstrate the relevance of malt selection not only for brewing but also for broader food innovation and health-oriented uses.

## 6. Nutritional Properties of Malted Cereals

Various processing technologies can alter the physicochemical composition of cereal grains. Malting is often cited for its potential to improve nutrient digestibility, as proteins and starches are partially hydrolyzed into simpler forms. However, the extent of improvement can vary with cereal type, cultivar, and malting conditions, and some apparent gains may reflect concentration effects due to moisture loss rather than absolute increases in nutrient content [39,40].

Studies on sorghum grist indicate that malting can alter both nutritional and antinutritional components. While reductions in tannins, phytates, trypsin inhibitors, and oxalates are reported, the magnitude of improvement depends on malting duration, temperature, and sorghum variety. Some observed increases in nutrient concentrations may partly result from dry matter loss rather than a true enhancement of absolute nutrient content [39,41,42].

Evidence for millet is more limited, but malting appears to improve enzymatic activity (α-amino nitrogen, α- and β-amylase) and other quality parameters. As with other cereals, responses vary by cultivar and malting protocol, and reported gains may partly reflect moisture loss rather than absolute nutrient enrichment [39,43].

Sprouting (germination) can enhance digestibility of protein and starch in cereals such as barley, rice, and maize [44,45] and reduce antinutritional factors in legumes [46,47]. Nutritional benefits in quinoa and other grains include higher antioxidant capacity and B-vitamin content [48,49]. However, these effects are highly dependent on germination conditions and cultivar, and some apparent improvements may result from concentration effects during water loss rather than absolute nutrient increases.

Malting may provide additional advantages over simple germination, producing dry, shelf-stable products such as malt flour that can be used in bread, snacks, and other foods. Nonetheless, the nutritional impact of these products depends on both the cereal type and the processing conditions, and improvements should not be assumed universal [44,45].

The effects of malting on the physicochemical characteristics of wheat and barley, as well as on the nutritional composition of the grains and their malts, have been investigated [50]. Their study examined changes in crude fiber, crude protein, and total carbohydrate content before and after malting, as shown in Table 4.

Reported increases in crude protein and total carbohydrates in wheat and barley malts largely reflect germination-driven concentration effects as moisture is lost, rather than absolute gains. While the higher fiber content of germinated barley may offer potential dietary benefits, these effects are variable across cultivars and malting regimes, emphasizing the need for cautious interpretation [51].

Recent studies (Table 5) indicate that malting can enhance enzymatic activity, digestibility, and bioactive compound formation, while reducing antinutritional factors [52,53,54,55,56]. However, these effects are highly context-dependent, influenced by cereal species, cultivar, and processing parameters. Reported increases in nutrient content may sometimes reflect concentration effects rather than absolute improvements, underscoring the importance of critical interpretation.

## 7. Health Effects and Biochemical Changes During Malting

Malting is the controlled germination of cereals, followed by heat treatment, which modifies grain composition and enhances digestibility [62,63]. During germination, endogenous enzymes are activated or newly synthesized, mobilizing starch, protein, and lipid reserves. These biochemical changes can lead to increased enzymatic activity, improved nutrient availability, and the formation of bioactive compounds [62], including phenolics and Maillard reaction products [63,64].

### 7.1. Biochemical Transformations

Germination and kilning induce a range of enzymatic and chemical transformations (Table 6). During germination, amylases, proteases, and lipases are activated, partially hydrolyzing starches, proteins, and lipids, while kilning promotes the formation of Maillard-derived compounds and phenolics, contributing to antioxidant potential and influencing color, flavor, and bioactive content. These biochemical modifications provide a mechanistic basis for the functional properties of malted cereals, but the magnitude of these changes depends on cereal type, cultivar, and malting conditions, and may differ between laboratory, animal, and human studies. In oats, malting-associated phytase activity may improve absorption of zinc and iron, while malted maize shows increased ferulic acid content, which contributes to antioxidant activity and may support bone health in experimental models [65,66,67].

### 7.2. Evidence of Health Effects

**Figure 3:**
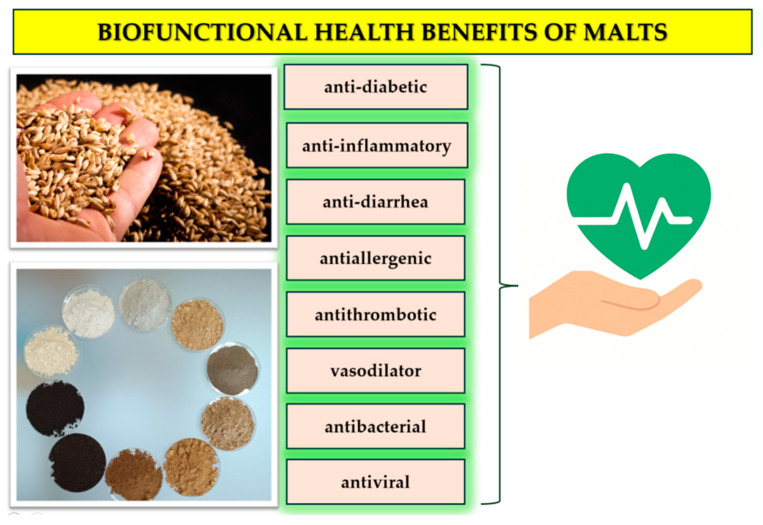
Health benefits of malts.

Several potential health effects have been reported for malted cereals (Figure 3, Table 6 and Table 7). It is important to distinguish between the types of evidence supporting each claim. Reported effects include antioxidant activity, modulation of glucose metabolism, gut protection, and mineral bioavailability (Table 6 and Table 7).

Animal studies have shown (Table 6) that malted barley extracts can reduce hyperglycemia and improve lipid profiles in genetically obese or diabetic mice, suggesting potential benefits for glucose regulation. During germination, enzyme activation mobilizes starch, proteins, and lipids, whereas kilning or roasting promotes the formation of phenolic and Maillard-derived antioxidants that support antioxidant, anti-inflammatory, and other physiological effects listed in Table 2 [62,63,68].

In vitro studies have demonstrated that malt-derived compounds, such as α-glucosidase inhibitors and phenolics, can influence glucose metabolism and antioxidant activity, but these findings do not directly predict human effects [65,76]. Animal studies are showing that malted barley extract can alleviate symptoms of diabetes in genetically obese mice, suggesting potential benefits for individuals with hyperglycemia or non-insulin-dependent diabetes mellitus [74,75,77]. Interest in the antidiabetic potential of barley malt has grown in recent years [67]. While promising, the translation of in vitro and animal effects to clinical outcomes remains uncertain, and results should not be overstated.

Research has examined the structure and activity of barley malt polysaccharides [76] and the effects of germination time on key components, including starch, β-glucan, β-glucanase, and α-amylase [77]. High α-glucosidase activity is associated with type II diabetes mellitus, as it can increase plasma glucose levels. Pure oligosaccharides from barley have shown in vitro inhibition of α-glucosidase, offering insights into the development of functional barley products [67].

Whole-grain cereals are associated with a reduced risk of metabolic syndrome, cardiovascular disease, and certain gastrointestinal cancers, in part due to their antioxidant phytochemicals, which may help mitigate inflammation. Malting may increase the bioavailability of these compounds, potentially enhancing their functional effects on metabolism and gut health; however, human evidence remains limited. Most evidence for gut-protective effects comes from in vitro or animal studies, and while these findings are promising, they cannot be directly extrapolated to prevention of gastrointestinal diseases in humans [78,79].

Studies also suggest that extensively malted wheat and oats can help prevent enterotoxic diarrhea and inflammatory bowel diseases such as Crohn’s disease and ulcerative colitis. This protective effect is attributed to phenolic compounds, including ferulic acid, vanillic acid, and guaiacol [78,79].

Germination and malting also increase polyphenol content in grains. Malted wheat, for example, shows higher polyphenol levels and biological activity than unmalted whole-grain wheat. Increased amylase activity during germination results in less starch and more sugars, potentially influencing the glycemic response [54].

Malting produces a range of biochemical transformations, including increased enzyme activity, β-glucan degradation, and phenolic content, which may collectively contribute to functional effects (Table 7).

Limited human trials indicate that consumption of germinated wheat or barley may improve postprandial glucose regulation and antioxidant status, although these studies are small and context-dependent [80].

The antioxidant potential of kilned and roasted malts is derived from both the natural compounds in barley and heat-induced transformations. Key contributors include polyphenols such as catechin, ferulic acid, coumaric acid, and various phenolic acids. Additional antioxidants, such as carotenoids, thiols, and vitamins, vary depending on the barley variety [68,81]. Research has demonstrated a strong correlation between antioxidant activity, malt color, and the biochemical composition of different malt types. As roasting intensity increases, both antioxidant capacity and browning also rise [82].

Malting also modifies other cereals and pseudocereals, such as buckwheat and millet, enhancing phenolic content, antioxidant activity, and mineral bioavailability. In buckwheat, for example, malting improves flavor and significantly increases phenolic content and antioxidant activity, particularly quercetin, vitexin, and orientin [83].

In oats, higher phytase activity during malting may enhance mineral bioavailability, potentially supporting gut health, although human evidence is limited. Naked oats, renowned for their high nutritional value, have been associated with potential benefits in reducing the risk of cardiovascular disease, diabetes, hypertension, and certain types of cancer. Enhancing their properties through malting may therefore support broader public health goals [84].

Millet malting can improve mineral bioavailability and shelf life, supporting its use in functional and infant foods. Germination enhances the bioavailability of minerals, including iron, zinc, and calcium, while also contributing to an extended shelf life [85]. Sorghum may serve as a gluten-free malted grain for food and beverage applications, with potential functional benefits demonstrated in vitro and in animal studies [86].

In summary, malting and germination modify cereal biochemistry, improving digestibility, bioactive content, and functional properties, although effects are context-dependent. These processes activate enzymes that break down starches, proteins, and lipids, improving the functional and nutritional properties of the resulting flours [87,88].

## 8. Food Safety and Mycotoxin Considerations in Malting

From a food safety perspective, the presence of certain fungi and the mycotoxins they produce poses a significant concern for cereal crops. Foods with higher fiber content, such as whole-grain products, may be particularly at risk, as parts of the grain (e.g., the husk) can accumulate mycotoxins [89].

Mycotoxins are toxic, low-molecular-weight secondary metabolites produced by fungi, especially molds. These include aflatoxins, ochratoxin A, fumonisins, trichothecenes, zearalenone, and patulin, predominantly from the *Aspergillus*, *Penicillium*, and *Fusarium* genera. Fusarium species alone are responsible for about one-third of all identified mycotoxins [90]. These toxins are thermally stable and can survive food processing, making them a persistent threat across the food supply chain from production to storage and consumption [91].

The toxicological effects of mycotoxins, known as mycotoxicosis, include hepatotoxicity, nephrotoxicity, immunosuppression, mutagenicity, and carcinogenicity. In Hungary, the most common mycotoxins include F2- and T2-toxins, deoxynivalenol (DON), ochratoxin A, alternariol, and alternariol monomethyl ether [91].

### 8.1. Regulatory Fragmentation and Harmonization Needs

Despite widespread acknowledgment of mycotoxin risks, significant regional disparities in food safety regulations persist, posing obstacles to effective risk management and international trade. Within the European Union (EU), maximum permissible levels of mycotoxins in cereals and cereal-based products are established under Regulation (EC) No. 1881/2006, with monitoring procedures outlined in Regulation (EC) No. 401/2006. Risk assessments are routinely conducted by the European Food Safety Authority (EFSA) [90]. In contrast, the United States Food and Drug Administration (FDA) sets “action levels,” often less stringent than EU limits. For example, aflatoxin concentrations are limited to 4 μg/kg in the EU, whereas up to 20 μg/kg is tolerated under FDA guidelines [90,92].

In many parts of Africa, Asia, and Latin America, comprehensive mycotoxin standards and surveillance systems remain underdeveloped [90]. This regulatory gap raises concerns regarding global health equity, particularly in tropical and subtropical regions where conditions are highly conducive to fungal contamination [93]. The lack of harmonized global standards hinders safe trade, consumer protection, and innovation in the valorization of malted cereals and brewer’s spent grain (BSG).

### 8.2. Analytical and Preventive Measures

No fully effective methods exist to completely eliminate mycotoxins from contaminated grains. However, preventive measures have significantly mitigated their occurrence throughout the production and post-harvest chain [94]. Preventive strategies include good agricultural practices (GAP) such as crop rotation, resistant cultivars, timely harvesting, and field hygiene [95]. Post-harvest handling techniques, namely, cleaning, dehulling, and storage under controlled conditions of temperature, humidity, and oxygen, have also been employed to inhibit fungal proliferation [96]. Mold development during storage is commonly managed through practices such as aeration, periodic turning of the stored grain, and regular quality inspections [97].

Analytical monitoring remains a cornerstone of mycotoxin risk management, with detection typically performed using techniques such as liquid chromatography–mass spectrometry (LC-MS/MS), enzyme-linked immunosorbent assay (ELISA), and other chromatographic methods [98]. Quantitative mycotoxin analysis is critical because the mere presence of fungal species does not necessarily indicate contamination [99].

### 8.3. Toward Harmonized Safety Standards in Malting

To facilitate the broader integration of malted cereals and brewer’s spent grain (BSG) into international food systems, the establishment of harmonized and enforceable safety standards for mycotoxins has increasingly been recognized as essential. It has been recommended that broader adoption of the Codex Alimentarius framework be pursued, particularly in emerging economies, where regulatory infrastructures may still be under development. Cross-border surveillance efforts are also encouraged, with coordination ideally led by institutions such as the European Food Safety Authority (EFSA), the Food and Agriculture Organization (FAO), the World Health Organization (WHO), and corresponding regional authorities [90].

To further promote regulatory alignment, the establishment of mutual recognition agreements between major trading blocs, such as between the European Union and the African Union (EU–AU), or the EU and Mercosur, has been proposed. In addition, the development and dissemination of low-cost, rapid diagnostic kits has been advocated to ensure that small-scale maltsters and producers incorporating BSG as a functional ingredient are adequately equipped to comply with safety standards [100].

Harmonization enhances consumer protection and fosters innovation in malted product use and by-product valorization, aligning with policies such as the European Green Deal and FAO’s Circular Bioeconomy Strategy.

## 9. Utilization Pathways for Brewer’s Spent Grain

BSG, made up of the outer layers and leftover endosperm of malted barley, is gaining recognition as a valuable raw material due to its high energy content and biotechnological potential [101]. Its growing importance in food and industrial applications is driven by its rich nutrient profile, low cost, and the increasing demand for sustainable ingredients. Compositional analysis shows that BSG contains significant amounts of carbohydrates (42–60% by weight), protein (14–24%), and dietary fiber (19–41%) [102]. Within its fiber content, hemicellulose (19–29%) and cellulose (16–33%) are the dominant components, while lignin levels range from 8% to 22%. BSG also contains lipids (3–10%) and a small amount of ash (1–4%), which reflects its mineral content [103].

**Figure 4:**
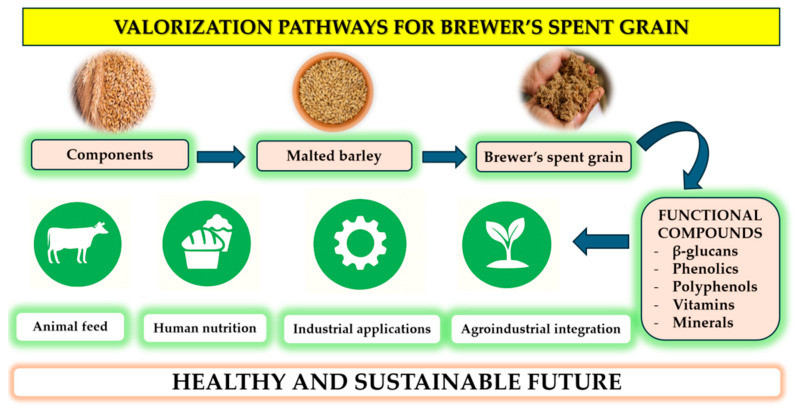
Multidimensional valorization pathways of brewer’s spent grain (BSG) toward a sustainable bioeconomy.

Figure 4 outlines the full valorization pathways for BSG, from its origin as a cereal component to its uses after brewing. BSG is rich in functional compounds, including phenolics, polyphenols, vitamins, and minerals, making it suitable for a diverse range of applications. These include use in animal feed, human food products, industrial processes [104], and agroindustrial applications such as biofertilizers and soil enhancers [105]. This multidimensional use of BSG highlights its role in supporting a circular bioeconomy, helping to reduce waste and build more sustainable, health-focused food systems.

Brewers’ spent grain (BSG) stands out nutritionally for its high content of β-glucans and phenolic compounds, both of which are linked to various health benefits [106]. Its protein profile is also notable, mainly due to its relatively high lysine content compared to other cereal by-products, making it a valuable addition to the human diet [107].

Even though BSG can contain up to 70% moisture, it still retains considerable amounts of polyphenols, with concentrations of around 212.85 mg GAE per kilogram, further enhancing its nutritional appeal [106]. In addition, BSG offers a wide range of essential vitamins, including biotin, choline, folate, niacin, pantothenic acid, riboflavin, thiamine, and pyridoxine. It is also rich in minerals like phosphorus, magnesium, calcium, silicon, iron, potassium, manganese, selenium, sodium, and sulfur [103].

Table 8 provides a comparative overview of the amino acid and mineral composition of BSG, malt, and raw barley, highlighting the key differences among these materials.

Recent studies have shed light on the impressive health potential of brewers’ spent grain (BSG), particularly its protein isolates. In cell-based experiments, these isolates significantly reduced the production of proinflammatory cytokines, indicating promising anti-inflammatory effects [110]. BSG has also been found to inhibit the angiotensin-converting enzyme (ACE), suggesting that it may help manage high blood pressure [109,111]. Additionally, phenolic compounds extracted from BSG have been shown to protect DNA from oxidative damage and inhibit enzymes involved in glucose metabolism, indicating a possible role in regulating blood sugar levels [112].

Animal studies further support these benefits. In obesity-induced rodents, diets enriched with BSG resulted in noticeable health improvements, including reduced body weight, enhanced insulin sensitivity, lower lipid levels, and increased production of beneficial short-chain fatty acids [113].

Thanks to its high fiber and protein content, BSG is now commonly found in human diets, most often as flour used in bread, cookies, pancakes, waffles, cakes, doughnuts, and other baked goods [114].

Why has barley historically been preferred for brewer’s spent grain (BSG) over other cereals? During the germination of barley, the cell walls of the endosperm are enzymatically broken down as the embryo develops and the rootlets, also known as chit. Barley grains anatomically have two-, three-, or even several-cell-thick aleurone cell layers, whereas all other cereals only have a single-cell-thick aleurone layer (Table 9). These cells contain characteristically protein-rich aleurone grains, which contain enzymes capable of hydrolyzing starch into smaller oligosaccharides and simple sugars. This special property, involving a multilayered aleurone cell band within the barley grains, results in the production and release of more enzymes. It translates into greater influence in converting starches into fermentable sugars during malting [103].

Using barley malt substitutes such as wheat, corn, and rice can result in longer saccharification times and higher viscosities, leading to prolonged wort separation associated with chemical problems in grain during malting (e.g., enzyme activation and content, starch hydrolysis, starch gelatinization temperature, protein hydrolysis, endosperm modification, density, pH) [117].

Malting’s final process, heating, results in the Maillard reaction. This process influences the flavor, aroma, color, and antioxidant properties of malted grains, making them more palatable and more beneficial for specific health conditions [60].

In addition, the use of brewer’s spent grain in food processing is known to have positive health effects and is expected to increase the food’s appeal. Adding BSG to products increases crumb firmness and sour flavor and can cause off-tastes and off-odors, thereby reducing overall acceptability in terms of appearance, texture, odor, mouthfeel, and taste [118]; among other effects, it can impart a sweet or bitter taste [115].

### 9.1. Potential for Feed Formulation

Brewer’s spent grain (BSG), a major by-product of the brewing process, is rich in fiber, protein, minerals, and bioactive compounds such as phenolic acids [104,119]. Its high moisture content (10–35%) makes it prone to spoilage, so preservation methods such as drying, ensiling, acidification, enzymatic hydrolysis, and microbial fermentation are commonly employed to improve digestibility, nutrient bioavailability, and reduce allergenic risks [120,121]. Traditionally used as animal feed, BSG is now attracting interest as a functional, hypoallergenic, and sustainable ingredient for both ruminant and non-ruminant diets [122].

#### 9.1.1. Animal Feed and Ruminant Nutrition

In ruminant diets, BSG serves as a cost-effective, fiber-rich supplement complementing forages like hay, silage, and straw [123,124]. Its fibrous structure, rich in non-starch polysaccharides, supports rumen fermentation and enhances nutrient uptake. Bioactive compounds such as phenolic acids may contribute to gut health and immunity, potentially reducing antibiotic use [104,119]. Ruminants can utilize BSG efficiently with minimal processing, though controlled drying or ensiling can preserve its nutritional quality and extend shelf life [22]. Globally, about 70% of BSG is used in animal feed, corresponding to roughly 27 million tons per year [120].

#### 9.1.2. Non-Ruminant Feed and Novel Ingredients

BSG is less digestible for monogastric animals such as pigs and poultry, and enzymatic treatments or microbial fermentation are often required to enhance nutrient absorption. Its fiber, residual yeast, and malt components improve satiety, support gut health, and boost growth performance when included with standard cereal feeds. In the EU alone, about 17 million tons of BSG are produced annually [22]. Beyond conventional feed, BSG is explored as a source of prebiotic fibers, protein isolates, and antioxidants [122], and in applications such as bioethanol production, bakery enhancement, and enriched animal feed formulations [125]. It also shows potential as a sustainable base for plant-based protein ingredients, offering an alternative to soy and peas [104]. Continued research focuses on safe storage, scalable processing, and functional applications in line with circular economy principles [126].

### 9.2. Industrial Applications: Bioplastics, Enzymes, and Bioactive Compounds

In addition to its use in food and animal feed, brewers’ spent grain (BSG) has growing value in industrial biotechnology and bioprocessing. On average, brewing generates 8–20 kg of BSG per hectoliter of beer, depending on the type of barley and brewing method used [35]. Although wet BSG contains about 90% water, the dry matter still holds much of the original grain’s composition, making it a rich source of lignocellulosic fibers, used in making paper, textiles, and biodegradable packaging [127], residual sugars and proteins ideal for enzyme production [35], and phenolic compounds known for their antioxidant and antimicrobial effects [128].

BSG has been successfully used as a raw material in various industrial processes. These include anaerobic digestion for biogas production, microbial fermentation to produce enzymes and organic acids, and bioplastic manufacturing, resulting in materials with good strength and antibacterial properties [127,129].

By turning BSG into valuable industrial products, companies can reduce waste, lower disposal costs, and support broader sustainability goals. These applications align well with low-carbon development strategies, promote resource efficiency, and contribute to the goals of the European Union’s Green Deal [100].

### 9.3. Agricultural and Agroindustrial Integration

BSG is closely tied to the broader goal of making cereal-based production more sustainable. Cereal farming naturally generates by-products, such as straw, bran, and husks, which are increasingly being utilized in biorefineries. Similarly, malting and brewing produce large amounts of fibrous leftovers that can be reintegrated into the cereal supply chain or used in new agroindustrial applications [22,130].

The malting process, which controls germination followed by kilning, not only turns grains into fermentable material but also leaves behind lignocellulosic residues [77]. These fibrous by-products can be recovered, stabilized, and repurposed through various sustainable pathways. These include: as biofertilizers using aerobic composting or solid-state fermentation [131] for soil improvement; applying partially digested husks and rootlets [132]; and as renewable feedstocks in biorefineries to produce biofuels, organic acids, and bioplastics [133].

Many smaller or regional malting plants, especially those affected by market consolidation or climate challenges, are currently underutilized. These facilities could be upgraded into decentralized, modular bioprocessing units. Doing so would enable local batch processing, improve logistics, and support rural circular economies by converting agricultural waste into new income streams [45,134].

By incorporating BSG and other cereal by-products into diverse agroindustrial systems, the sector can help build climate-resilient food systems, reduce biomass waste, and move toward a low-carbon, resource-efficient bioeconomy.

### 9.4. Practical Applications and Case Studies of BSG

The successful reuse of brewers’ spent grain (BSG) has been proven in many real-world cases across Europe, Latin America, and Asia. These examples show that it is entirely feasible to integrate BSG into food, animal feed, and bioindustrial applications on a commercial scale. Table 10 provides an overview of the main valorization pathways, outlining their uses, key advantages, and the primary challenges associated with each approach.

## 10. Challenges and Future Perspectives

Brewer’s spent grain (BSG) and malting by-products hold considerable promise for sustainable food production, yet several technical, economic, and regulatory challenges must be addressed to achieve optimized utilization [144].

Before addressing the remaining bottlenecks, it is essential to compare the available processing routes that determine the future valorization potential of BSG (Table 11 and Table 12). Malting is an effective biochemical process for reducing antinutritional substances to acceptable levels (Table 11). In addition, enzymatic treatment is an effective process that can be used to assist in the purification process [103].

One effective method is to combine different treatments to enhance the properties of brewer’s spent grain, as shown in Table 12. Enzymatic treatments can be coupled with microbial fermentation to produce energy-rich biofuels or other co-products, such as microbial lipids and carbohydrates [103]. Addition of Brewer’s Spent Grain with fermentation as a pretreatment shows promising outcomes due to the enrichment of nutrition and improves the textural properties [118].

Challenges include the scalability and market acceptance of BSG, as well as maintaining constant quality and safety requirements. A key paradigm for promoting sustainability in the grain processing value chain is the adoption of circular economy principles, which incorporate the processes of reducing, reusing, and recycling [147].

Infrastructure and processing limitations: Most existing malting and brewing facilities were not designed for BSG recovery or on-site processing. Retrofitting plants to include drying, stabilization, or microbial control often requires substantial investment and additional space. Energy-efficient drying technologies, such as solar-assisted, infrared, and hybrid systems, have demonstrated the ability to preserve valuable compounds while reducing energy consumption; however, their adoption remains limited, particularly in small-scale breweries, due to upfront costs and operational complexity. Similarly, microbial fermentation using lactic acid bacteria or selected yeasts can extend shelf life and reduce spoilage, but successful implementation requires careful strain selection, process monitoring, and compliance with food safety regulations [148].

Innovative solutions and digital integration: Modular, container-based processing units offer a potential solution for localized BSG handling, particularly for small maltsters and craft breweries. Early implementations suggest these units can maintain product quality and facilitate decentralized processing, but comprehensive evaluations of scalability, cost-effectiveness, and energy efficiency are still limited. Digital tools, including IoT-based sensors, AI-driven monitoring, and blockchain for traceability, have been proposed to improve consistency and support sustainability. While promising, these approaches require rigorous validation under real-world brewery conditions [100,149,150].

Consumer perception and market barriers. Despite its high nutritional and environmental value, BSG is frequently regarded as “waste,” hindering market acceptance. Effective strategies to overcome this perception include targeted consumer education, clear product labeling, and product design emphasizing health benefits and eco-friendly attributes [151]. However, quantitative studies on consumer willingness to pay for BSG-enriched products remain scarce, representing a critical knowledge gap.

Regulatory and standardization challenges: Variability in food safety and quality regulations complicates cross-border trade and large-scale commercialization. Harmonization of standards, through frameworks such as Codex Alimentarius or mutual recognition among regional authorities, would facilitate broader adoption of BSG-based products while ensuring safety and consistency [152].

Climate and environmental considerations: Climate change poses additional risks by affecting cereal yields, malt quality, and the likelihood of fungal contamination. Strategies under investigation include the development of climate-resilient cereal varieties, predictive mycotoxin monitoring, and adaptable processing technologies capable of maintaining product quality under variable environmental conditions [153].

Future directions and integration: Advances in food biotechnology, fermentation, and drying technologies continue to expand the potential applications of both malted cereals and BSG. Collaborative efforts among academia, industry, and policymakers, aligned with initiatives such as the European Green Deal, FAO Circular Economy Strategy, and UN Sustainable Development Goals, are critical for accelerating the translation of research into practice [154]. Integrating malting and BSG reuse into cereal value chains can transform conventional, linear processing into a circular system, enabling climate-smart agriculture, waste reduction, and enhanced nutritional outcomes.

**Figure 5:**
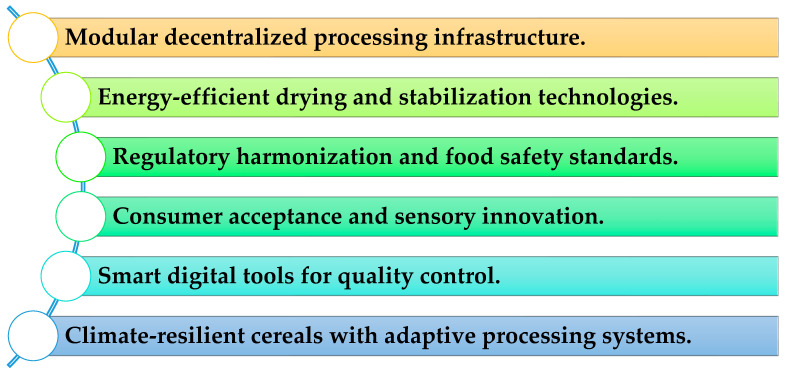
Critical pathways for next-generation R&D.

In conclusion, while technical and socioeconomic barriers remain, evidence-based strategies focusing on infrastructure optimization, innovative processing, consumer acceptance, regulatory alignment, and climate resilience are essential to realize the full potential of BSG as a sustainable food ingredient. The pathways for next-generation development are presented in Figure 5.

## 11. Conclusions

Cereal crops have long supported human civilization, providing essential nutrients. Malting, a processing technique refined over centuries, blends tradition with innovation. Through carefully managed germination and drying, natural enzymes are activated, nutrient absorption is enhanced, and the production of functional compounds with antioxidant, anti-inflammatory, and antidiabetic properties can be stimulated, benefiting human health. The brewing industry generates a substantial by-product, brewer’s spent grain (BSG), which remains underutilized despite its nutritional richness. BSG retains significant amounts of protein, fiber, and polyphenols, making it a promising ingredient for future circular food systems. Technological advances in drying, stabilization, and safety assurance will be key to unlocking its full potential.

International collaboration among researchers, policymakers, and industry stakeholders is essential to realize these opportunities. Malting, therefore, is more than a method of food production; it is an ancient craft and a modern tool for building a sustainable, nutritious future.

## Figures and Tables

**Table 1:** Tritordeum malt analysis provided by Agrasys (source: [16]).

Parameter *	Result	Unit	Method
Moisture content	5.5	%	EBC 4.2
Saccharification time	10–20	Min	EBC 4.5.1/4.5.2
Filtration time	<1	H	
Extract dry matter	84.1	%	
Smell	Normal		
pH	5.77		
Color EBC	4.13	EBC	EBC 4.7.1
Viscosity	1.45	mPas·s	EBC 4.8
Total Protein	12.0	%	EBC 4.3.1
Soluble Protein	6.2	%	EBC 4.9.1
Diasteic powder	449	WK	EBC 4.12.1
Kolbach Index	54	%	MEBAK 3.1.4.5.3

* All analyses were performed in duplicate.

**Table 2:** Barley malt analysis provided by Ireks (source: [17]).

Parameter	Standard Specification	Unit	Method *
Moisture Content	<5	%	R-200.18.020 [2016-03]
Extract, dry matter	>80	%	R-205.01.080 [2016-03]
Wort Color	2.5–5	EBC	R-205.07.731 [2016-03]
Boiled Wort Color	4–7	EBC	R-205.08.110 [2016-03]
Saccharification time	5–15	Min	R-205.03.730 [2016-03]
Soluble Nitrogen, dry matter	610–780	mg/100 g	R-205.11.030 [2016-03]
Protein Content, dry matter	9.0–11.5	%	R-200.20.030 [2016-03]
Kolbach Index	36–45	%	R-205.12.999 [2016-03]
pH value	5.8–6.1		R-205.06.040 [2016-03]
FAN (Trs.)	110–160	mg/100 g	R-205.14.111 [2016-03]
Viscosity (congress wort at 8.6%)	<1.58	mPas·s	R-205.10.282 [2016-03]
Viscosity (Iso 65 °C at 8.6%)	<1.65	mPas·s	R-205.10.282 [2016-03]
β-Glucan (congress wort)	<300	mg/L	R-205.15.111 [2016-03]
β-Glucan (Iso 65 °C)	<400	mg/L	R-205.15.111 [2016-03]
Friability	>81	%	R-200.14.011 [2016-03]
Compl. Glassy	<2.5	%	
Dimethyl Sulfide-Prec. (DMS-P) dry matter	<7	mg/kg	R-205.29.153 [2016-03]
Congress wort			R-206.00.002 [2016-03]
Iso 65 °C			R-207.00.002 [2016-03]

* Analysis method according to MEBAK, Raw Materials, 2016 [18]. Brewing grain is a natural product and is subject to qualitative fluctuations, which can influence the specification values listed.

**Table 3:** Some scientific findings on malt development and enzymatic properties.

Research Focus	Key Findings	Relevance to the Topic	Ref.
Metabolomic profiling of malt during the malting process	LC-MS and GC-MS revealed increased levels of amino acids and Maillard intermediates during kilning, correlating with color and flavor formation.	Explains how kilning temperature drives chemical reactions responsible for flavor and color in specialty malts (e.g., Vienna, Munich, crystal).	[36]
Enzymatic gluten-hydrolysis in beer production using prolyl endopeptidase (AN-PEP)	Prolyl endopeptidase (AN-PEP) reduced gluten in beer < 20 mg/L without changing color or taste.	Highlights the biotechnological use of malt-derived enzymes and the relevance of enzymatic activity (high in pale malts, absent in dark roasts).	[37]
Germination duration and enzymatic activity in barley malt cultivars	Longer germination increased α- and β-amylase activity, as well as protease activity, which affects protein balance and haze formation.	Connects malt modification level to enzymatic activity; explains why pale malts are well-modified while Munich or dark malts are not.	[38]

**Table 4:** Chemical composition of wheat, barley, wheat malt, and barley malt (source: [51]).

Grains	Wheat	Wheat Malt	Barley	Barley Malt
Moisture %	12.9	14.54	12.6	7.13
Ash %	1.8	1.02	2.1	1.95
Crude protein %	12.3	14.19	11.06	11.76
Total carbohydrates %	67.8	79.42	63.5	65.43
Total fat %	1.89	1.73	1.91	1.27
Crude fiber %	2.11	1.65	9.9	8.15

**Table 5:** Recent scientific findings on the nutritional and functional effects of malting (2023–2025).

Type of the Study	Main Findings	Relevance	Ref.
Barley and Malt as Base Ingredients for the Production of New Bio-Functional Foods.	Malting increased amino acids, phenolic compounds, and antioxidant activity in barley.	Confirms improved nutritional and bioactive profile.	[57]
Malting of Barley and Wheat Grains Impacts Their Metabolic Profiles in a Model of In Vitro Colonic Fermentation	Malted barley and wheat showed altered metabolite profiles and higher amino acid availability during digestion.	Indicates enhanced digestibility and nutrient release.	[58]
Emerging approaches to improve barley malt processing	Emerging technologies (e.g., ultrasound pre-treatment) improved enzyme activation and starch conversion.	Demonstrates the potential of modern malting optimization.	[59]
The role of malting in grains and legumes for improved nutritional and functional value	Malting effectively reduced antinutritional factors and increased bioactive compounds.	Highlights the nutritional and functional advantages of malting.	[60]
Optimization of Millet Malting Parameters Using Artificial Intelligence-Based Modelling	AI-optimized malting improved enzyme activity and diastatic power in millet.	Expands malting potential to underutilized cereals.	[61]

**Table 6:** Reported health benefits of malted cereals.

Health Effect	Mechanism/Key Compound	Example Grain	Refs.
Antidiabetic	α-glucosidase inhibition, enhanced glucose uptake	Barley, wheat	[52,53]
Antioxidant	Phenolic acids (ferulic, vanillic, guaiacol), tocopherols, vitamin C	Barley, wheat, buckwheat	[54,55,68]
Anti-inflammatory	Phenolic and flavonoid fractions	Maize, oats	[56,63]
Cholesterol-lowering	Glucose tolerance factor	Barley	[52]
Gut protection	Phenolic leachates and antioxidants	Oats, wheat	[65,69]
Bone protection	Ferulic acid	Maize	[66]

**Table 7:** Comparative effects of malting on different cereal grains.

Cereal	Effect of Malting	Major Bioactive Compounds	Health Relevance	Ref.
Barley	Enhanced enzyme activity, β-glucan degradation	Phenolic acids, carotenoids	Blood glucose control, antioxidant action	[70]
Wheat	Higher polyphenol and antioxidant levels	Vanillic acid, tocopherol	Improved glycemic regulation	[67]
Oat	High phytase activity → better Fe and Zn absorption	Phenolics, phytase	Gut protection, mineral bioavailability	[71]
Maize	Rich in ferulic acid	Ferulic acid	Antidiabetic, antioxidant, and bone protection	[72]
Buckwheat	Strongly increased polyphenols	Quercetin, vitexin, orientin	Antioxidant, flavor improvement	[73]
Millet	Improved Fe, Zn, Ca bioaccessibility	Polyphenols	Infant foods, functional foods	[74]
Sorghum	Gluten-free, suitable for fermentation	Phenolic compounds	Diverse food applications	[75]

**Table 8:** Amino acid ^a^ and mineral content of BSG, malt, and barley (source: [108,109]).

		Tested Samples		
Tested Parameters		BSG	Malt	Barley
Non-essential amino acids	Histidine	26.27	1.90	1.59
	Glutamic acid	15.59	0.75	0.85
	Aspartic acid	4.81	0.17	0.19
	Valine	4.61	0.24	0.23
	Arginine	4.51	0.23	0.21
	Alanine	4.12	0.23	0.22
	Serine	3.77	0.07	0.12
	Tyrosine	2.57	0.14	0.14
	Glycine	1.74	0.06	0.08
	Asparagine	1.47	0.33	0.23
	Glutamine	0.07	n.d.	n.d.
Essential amino acids	Lysine	14.31	3.69	2.52
	Leucine	6.12	0.29	0.30
	Phenylalanine	4.64	0.21	0.20
	Isoleucine	3.31	0.17	0.17
	Threonine	0.71	0.02	0.01
	Tryptophan	0.14	n.d.	0.01
	Methionine	n.d.	n.d.	0.03
Mineral content (% *w*/*w*)	Phosphorus	0.46	0.27	0.24
	Magnesium	0.24	0.09	0.08
	Calcium	0.22	0.05	0.06
	Silicon	0.14	0.06	0.05

^a^ Expressed as a percentage of total. n.d. explained as “no data”.

**Table 9:** A comparison of the nutritional properties of brewer’s spent grain and other cereal by-products.

Cereal Sources	By-Product	Processability	Nutritional Properties/ Bioactive Components	Application Potential	References
Barley	Brewers’ spent grain	two-, three-, or even several-cell-thick aleurone cell layers	stable, nutrient-rich composition; water holding capacity; lowering the glycemic index; bioavailability; rich in dietary fiber, protein, vitamins, minerals, bioactive compounds	in functional foods; nutraceuticals; bio-based materials, animal feed, biomass energy production	[103,115,116]
	flour from bran		β-glucans, soluble and insoluble arabinoxylans	in functional foods; gut-health formulation	
Wheat	bran	Single-cell-thick aleurone	antioxidants, dietary fiber, protein	baked goods, dietary fiber supplements	[103,116]
	germ		proteins, essential amino acids, ferulic acid, β-glucans	energy-dense products, nutraceuticals	
Corn	bran		fiber, antioxidants, proteins	fortified bakery products	
	germ		oil, proteins, phytochemicals	nutraceuticals, oil extraction	
Rice	bran		phenolic compounds, antioxidants	natural preservatives, supplements	
	germ		lipids, phytosterols	functional oil	
Oats	husk		phenolic acids, β-glucans	dietary supplements	
Sorghum	bran		tannins, flavonoids	functional foods, bioethanol	

**Table 10:** Valorization pathways for brewer’s spent grain (BSG).

Valorization Pathway	Applications	Key Benefits	Main Challenges	Refs.
Animal Feed (Ruminants)	Silage supplement, forage extender	High fiber improves rumen fermentation and has a low cost	Moisture instability, antinutritional compounds	[135]
Non-Ruminant Feed	Poultry, pigs, and aquaculture feed	Protein source, gut health, enzyme co-feed	Low digestibility, needs processing	[136,137,138]
Functional Food Ingredient	Bread, cookies, pancakes, snacks	High fiber, protein, polyphenols, prebiotics	Flavor masking, moisture control	[115]
Bioethanol and Biogas	Renewable fuel via fermentation or anaerobic digestion	Waste reduction, energy recovery	Infrastructure costs, fermentation inhibitors	[139]
Bioplastics	PLA composites, packaging materials	Biodegradable, carbon-neutral material	Mechanical strength, scale-up	[140]
Enzyme Production	Fermentation media for amylase, protease, and lipase	Low-cost nutrient-rich substrate	Microbial optimization	[141]
Phenolic Extraction	Antioxidants for cosmetics, nutraceuticals	High-value bioactives, oxidative protection	Solvent choice, extraction efficiency	[134]
Agroindustrial Integration	Biofertilizers, soil amendments, biorefinery feedstocks	Circular agriculture, local resource loops	Logistics, storage, market access	[142]
Case-Specific Solutions	Localized drying, modular reuse, bakery linkage	Custom strategies, community engagement	Varies by region and tech availability	[143]

**Table 11:** Comparative evaluation of malting and alternative cereal processing technologies in terms of nutritional functionality and sustainability.

Different Treatments	Nutritional Effects	Techno-Functional Effects	Sustainability/ Limitations	References
Malting	Reduces antinutritional factors (disulfide bonds, hordeins); increases fiber; enhances bioavailability; increases polyphenols and GABA	Improved oil-binding capacity; better functional properties of flours	Biochemical, mild process suitable for special-diet products	[60,103,106]
Enzymatic treatment	Increases lignans (syringaresinol, secoisolariciresinol); improves protein digestibility	Higher protein solubility; improved foaming and emulsifying ability	Requires enzyme inputs; industrially scalable	[103,118,145,146]
Fermentation	Reduces antinutritional factors; increases protein digestibility; releases free amino acids; improves mineral bioavailability (Fe, Ca, Zn)	Improves sensory attributes and bioactivity	May cause nutrient leaching and off-flavors; allows for valorization of low-value by-products, reducing production cost	[60,116,145]
Sprouting	Improves blood pressure regulation and metabolic health	Enhances enzymatic activity in grains	Low-cost, natural bioprocess	[60]
Soaking	Removes water-soluble antinutrients (tannins, oxalates); improves mineral and vitamin B12 absorption	Simple pre-treatment step	May cause nutrient losses into soaking water	[60]
Thermal processing	Effectively reduces antinutrients	-	High risk of nutrient loss due to heat degradation	[60]

**Table 12:** Comparative analysis of different treatments for enhancing BSG properties [118].

Different Combinations of Treatments	Effect
Thermal and enzyme treatment	Treatment increases the free phenolic content
Alkaline and enzyme treatment	Direct enzymatic hydrolysis of BSG without alkaline treatment is more beneficial
Solid-state fermentation	Fermentation improves the nutrient content of BSG can be observed by changes in metabolites amino acids, citric acid, vitamins, and antioxidants.
Hydromechanical processing	Generating rich protein extract with stabilizing properties.
Pulsed electric field treatment	The treatment increases the yield of total free and bound phenolic compounds.
Combination of enzyme and fermentation	Bioprocessed BSG obtains enhanced biological activity; novel antioxidant peptides are observed.

## Data Availability

No new data were created or analyzed in this study. Data sharing is not applicable to this article.
